# Pentafuhalol-B, a Phlorotannin from Brown Algae, Strongly Inhibits the PLK-1 Overexpression in Cancer Cells as Revealed by Computational Analysis

**DOI:** 10.3390/molecules28155853

**Published:** 2023-08-03

**Authors:** Waseem Ahmad Ansari, Safia Obaidur Rab, Mohammad Saquib, Aqib Sarfraz, Mohd Kamil Hussain, Mohd Sayeed Akhtar, Irfan Ahmad, Mohammad Faheem Khan

**Affiliations:** 1 Department of Biotechnology, Era’s Lucknow Medical College & Hospital, Era University, Sarfarazganj, Hardoi Road, Lucknow 226003, India; ansariwasim144@gmail.com (W.A.A.);; 2 Department of Chemistry, Era University, Sarfarazganj, Hardoi Road, Lucknow 226003, India; 3 Department of Clinical Laboratory Sciences, College of Applied Medical Sciences, King Khalid University, Abha 62529, Saudi Arabia; srab@kku.edu.sa (S.O.R.);; 4 Department of Chemistry, University of Allahabad, Prayagraj 211002, India; saquibem@gmail.com; 5 Department of Chemistry, Government Raza P.G. College, Rampur, M. J. P. Rohilkhand University, Bareilly 244901, India; mkhcdri@gmail.com; 6 Department of Botany, Gandhi Faiz-e-Aam College, Shahjahanpur 242001, India

**Keywords:** brown algae, Pentafuhalol B, PLK-1, cancer, molecular docking, molecular dynamic simulation, DFT

## Abstract

Polo-like kinase-1 (PLK-1) is an essential mitotic serine/threonine (Ser/Thr) kinase that belongs to the Polo-like kinase (PLK) family and is overexpressed in non-small cell lung cancer (NSCLC) via promotion of cell division. Therefore, PLK-1 may act as a promising target for the therapeutic cure of various cancers. Although a variety of anti-cancer drugs, both synthetic and naturally occurring, such as volasertib, onvansertib, thymoquinone, and quercetin, are available either alone or in combination with other therapies, they have limited efficacy, especially in the advanced stages of cancer. To the best of our knowledge, no anticancer agent has been reported from marine algae or microorganisms to date. Thus, the aim of the present study is a high-throughput virtual screening of phlorotannins, obtained from edible brown algae, using molecular docking and molecular dynamic simulation analysis. Among these, Pentafuhalol-B (PtB) showed the lowest binding energy (best of triplicate runs) against the target protein PLK-1 as compared to the reference drug volasertib. Further, in MD simulation (best of triplicate runs), the PtB-PLK-1 complex displayed stability in an implicit water system through the formation of strong molecular interactions. Additionally, MMGBSA calculation (best of triplicate runs) was also performed to validate the PtB-PLK-1 complex binding affinities and stability. Moreover, the chemical reactivity of PtB towards the PLK-1 target was also optimised using density functional theory (DFT) calculations, which exhibited a lower HOMO-LUMO energy gap. Overall, these studies suggest that PtB binds strongly within the pocket sites of PLK-1 through the formation of a stable complex, and also shows higher chemical reactivity than the reference drug volasertib. The present study demonstrated the inhibitory nature of PtB against the PLK-1 protein, establishing its potential usefulness as a small molecule inhibitor for the treatment of different types of cancer.

## 1. Introduction

Phlorotannins, abundantly distributed in brown algae, are an important class of polymeric phloroglucinol containing multiple hydroxyl groups, which makes them highly soluble in water. They are polymerised through shikimate pathways via connecting C-C or C-O-C linkages [1]. Based on types of linkages, phlorotannins are divided into different groups such as fucols (phenyl bond), phlorethols (ether bond), fucophlorethols (ether and phenyl bond) eckols, fuhalol, and carmalols (a dibenzo-dioxin bond). The hydroxyl group is present in eckols and fuhalols, but eckols can be distinguished by the 1,4-dibenzodioxin structure, whereas carmalols are made up of phlorethol moieties that, like eckols, contain hydroxyl groups [2]. Most of the phlorotanins are found in golden-brown algae, which comprise a diverse class of the Pheophyceae family of giant complex seaweeds [3,4,5]. Phlorotannins show specific biological activities such as antioxidant, anti-inflammatory, anti-cancer, anti-diabetic, and anti-viral properties. Brown algae such as Ecklonia are well known for their antidiabetic, antioxidant, and anti-cancer, activities because they contain different types of phlorotanins, namely eckol, dieckol, phloroglucinol, phlorofucofuroeckol A, and fucodiphlorethol G [6]. Edible brown algae, namely Eisenia contain phlorofucofuroeckol B which exhibits anti-allergic activity [7]. Phloroglucinol isolated from *Himanthalia elongate* demonstrated promising anti-microbial and antioxidant activities [8]. Some studies revealed that phlorotannins may act as antiviral agents by inhibiting human papillomavirus (HPV16PVs and HPV18PVs), influenza A like H1N1 and H9N2, human immunodeficiency virus type-1 (HIV-1), viral hemorrhagic septicemia virus (VHSV), murine norovirus (MNV), as well as coronaviruses [9]. An in vitro study showed that a phlorotannins-rich extract of brown seaweed, namely *Saccharina japonica* Aresch, inhibited the growth of human hepatocellular carcinoma cells (HepG2). This anticancer activity was due to enhanced cell death by inducing the apoptosis process [10]. Phloroglucinols obtained from brown algae *Costaria costata* have been shown inhibitory effects against α-NaGalases enzyme, which is found in HuTu 80 and SK-MEL-28 cells. The IC_50_ values were noted as 0.14 ± 0.008 and 0.12 ± 0.004 mg/mL for both the cancer cell lines, respectively [11]. Eckstolonol and phlorofucofuroeckol-A, containing a fraction of *Fucus vesiculosus* algae, inhibited the growth of Caco-2 colorectal and MKN-28 gastric cancer cells by showing cell death through apoptosis and necrosis cascade. Additionally, they inhibited cell proliferation against various cancer cell lines, including Caco-2, HT-29, BEL7402, mouse leukemia (P388), and mouse teratocarcinoma (ATDC5) [12]. However, the inhibitory role of phlorotannins against Polo-like kinase-1 (PLK-1), a serine/threonine-protein kinase enzyme that regulates the cell cycle, is still unknown and needs to be explored. Further, PLK-1 has emerged as a key regulator of progressing events during mitosis, such as the G2/M transition, spindle formation, and segregation of chromosomes, which take part in cell proliferation processes [13]. A failure in cell cycle regulation produces various problems in metaphase by favouring aberrant cell survival, which results in aneuploidy and genetic instability inside the cells, followed by cancer [14]. Overexpression of PLK-1 has been found in various types of cancers such as squamous cell carcinomas, non-small cell lung cancer, oropharyngeal cancer, and ovarian and endometrial cancers via the inactivation and/or degradation of the tumour suppressor gene p53 in a G2- and S-phase-expressed state. PLK-1 overexpression also increases the centrosome size and/or number, which will lead to improper segregation of chromosomes, aneuploidy, and tumorigenesis. PLK-1 can inhibit the transactivation and pro-apoptotic functions of p53 function through physical interaction and phosphorylation [15,16]. Thus, it has been proposed that the expression of PLK-1 could be used as a therapeutic target for various types of cancers.

Over the past few decades, the design and development of alternative therapeutic molecules from biological sources have been a long-term strategy to strengthen the drug development process for a variety of human diseases and ailments [17]. Currently, numerous techniques are available for screening and predicting promising therapeutic agents against different targets in various diseases. In this context, computational tools comprising molecular docking, MD simulation, and density functional theory (DFT) studies have gained significant attention for studying the molecular interactions of ligands with target macromolecules as well as their chemical reactivity [18]. Despite the advancements in newer techniques for the screening of drug molecules, cancer remains the deadliest among the numerous diseases and ailments. The slow rate of screening, various side effects, and complex genetic trees are the most common failures in the treatment of cancers [19]. These hurdles can be removed by employing molecular docking and simulation methods to recognise the best ligand against each receptor target in the process of drug design and development. Undoubtedly, polyphenols, for example, including phlorotannins, have been extensively studied by using computational tools against several diseases and ailments by targeting numerous molecular signaling pathways. Gunaseelan et al. identified many phlorotannins as alternative therapeutic agents against SARS-CoV-2 replicative proteins through molecular docking and MD simulation analysis using AutoDock Vina 1.5.6 version. They found that phlorotannins were involved in interacting with the Glu166, Gln189, Cys145, and Thr190 tetrad amino acid residues to inhibit 3CLpro activity [9]. Bakunina et. al. showed that phlorotannins, namely DP-15 oligophlorethol and heptaphlorethol obtained from brown algae *Costaria costata*, act as inhibitors through interactions with amino acid residues (Asp 156 and Asp 217) of human α-NaGalase in cancer cells [11].

Based on previous literature, we crucially feel that phlorotannins may play key roles in combating cancer as well as other diseases by developing them as lead therapeutic agents with minimal or no side effects. Keeping these barriers in mind, our study aimed to identify the phlorotannins, compounds from edible brown algae, as therapeutic agents against the receptor protein (PLK-1) that is involved in mitotic cell division in the cell proliferation processes of cancer cells. PLK-1 is well known to express an oncogenic effect in various types of cancer [20]. PLK-1 controls the mitosis stage during cell division, and any dysfunction in PLK-1 regulation leads to cancer cell progression [13]. Thus, our study could be used to determine the potential leads against the development of different cancers.

## 2. Results and Discussions

### 2.1. Structure Optimisation by DFT Study

**Figure 1:**
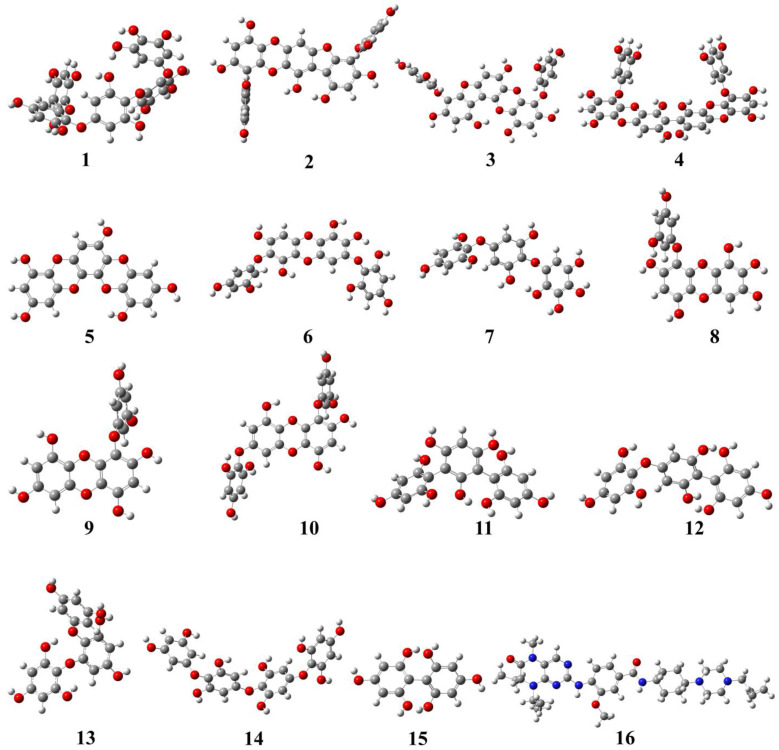
Geometrically optimised structures of phlorotannins (**1**–**15**) as well as the control drug (**16,** volasertib) calculated by the B3LYP hybrid function combined with a 6–31 basis set using the DFT method.

**Figure 2:**
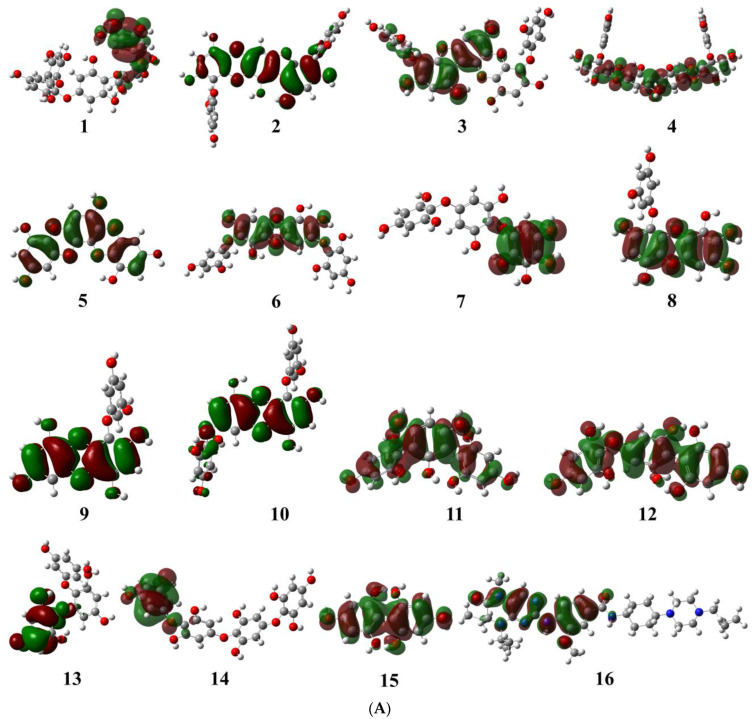

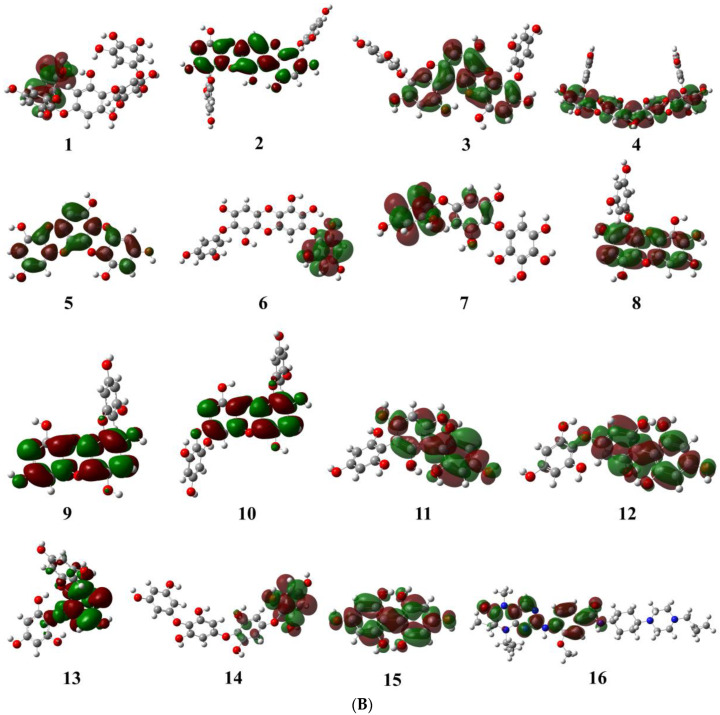
(**A**) Pictorial representation of HOMO orbitals for phlorotannins (**1**–**15**) and the control drug (**16**). (**B**) Pictorial representation of LUMO orbitals for phlorotannins (**1**–**15**) and the control drug (**16**).

**Figure 3:**
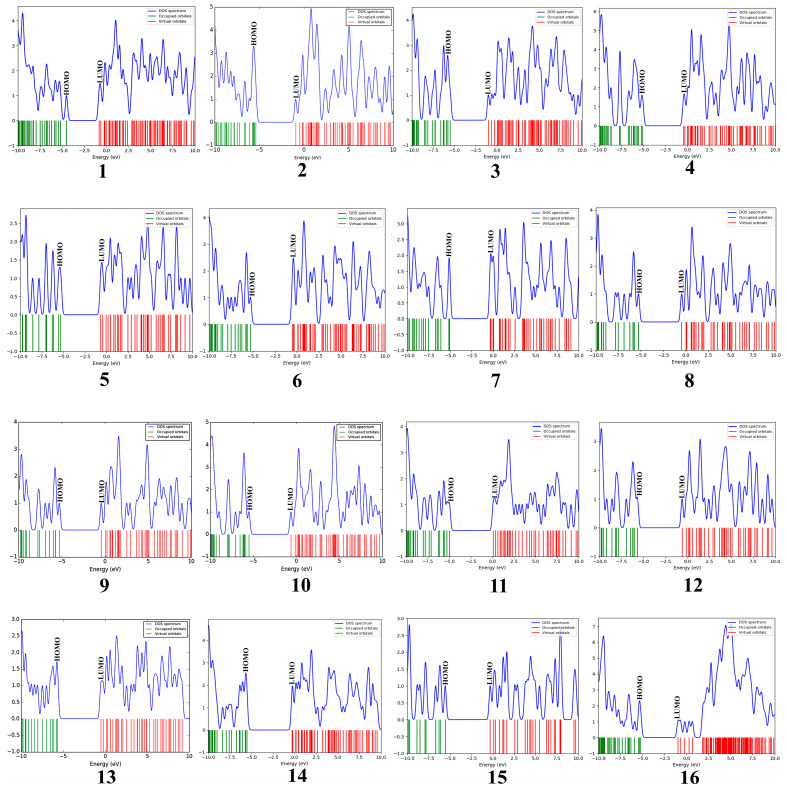
Density of states (DOS) plots of phlorotannins (**1**–**15**) and the control drug (**16**).

**Figure 4:**
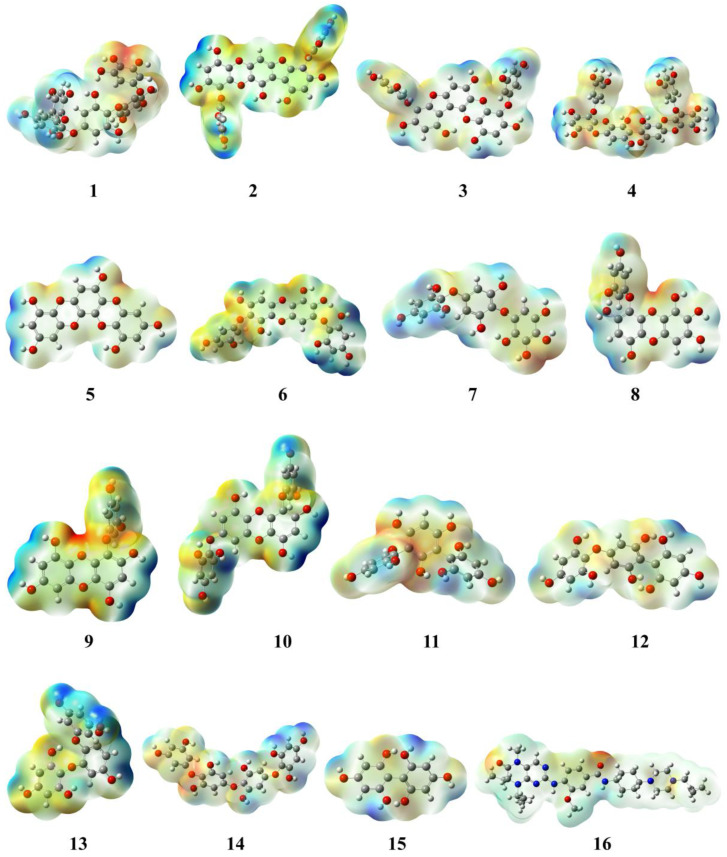
Molecular electrostatic potential (MEP) map of phlorotannins (**1**–**15**) and control drug (**16**).

The geometrical structures of phlorotannins (**1**–**15**) and control drugs such as volasertib (**16**) were optimised by the B3LYP hybrid function with a 6–31 G basis set using the DFT method (Figure 1). DFT is the most commonly used computation based on quantum theory to optimise the electrical structures of isolated atoms or molecules. To describe the mechanism of action at enzyme sites during drug-receptor interactions, it offers the chemical correctness of drug molecules. In DFT, the frontier molecular orbitals such as HOMO and LUMO play an important role in estimating the chemical reactivity (electronic properties) of the compounds. Similarly, the energy gap (E_gap_) between HOMO and LUMO is associated with the reactivity and stability of the compounds through the enhancement of intramolecular charge transfer within the compounds. The other quantum descriptors or parameters were also studied to ascertain which functional group of the compound is good for electronic properties [21]. These descriptors were comprised of ionisation energy (IE), electron affinity (EA), hardness (ƞ), and softness (σ), which are involved in defining the chemical properties of a compound [22]. The calculated HOMO-LUMO gap, chemical descriptors, density of states (DOS), and molecular electrostatic map of all the selected phlorotannins and volasertib (**1**–**16**) are displayed in Figure 2, Figure 3 and Figure 4 as well as Table 1.

#### 2.1.1. Evaluation and Analysis of Frontier Molecular Orbitals

The molecular orbital energy calculations of HOMO (highest occupied molecular orbital) and LUMO (lowest unoccupied molecular orbital) are used to define the chemical reactivity and stability of the compounds (Figure 2A,B). The HOMO orbitals work as electron donors whereas the LUMO orbitals act as electron acceptors. The space between the HOMO and LUMO is known as the HOMO-LUMO gap, which calculates intermolecular electron transfer within the molecule [23]. Moreover, the low energy gap between HOMO and LUMO implies less stability and more reactivity; on the other hand, the large energy gap between HOMO-LUMO indicates less reactivity and more stability. The HOMO energy of the phlorotannin compounds was evaluated from −4.513 eV to −5.570 eV, while the LUMO energy was from −0.810 eV to −0.307 eV, whereas volasertib exerted the −5.192 eV HOMO energy to transfer an electron; the −0.970 eV energy was required to accept an electron for the LUMO orbital (Table 1). In our study, among the phlorotannins, PtB exhibited the lowest energy gap (E_gap_ = 3.703 eV), calculated from HOMO (E_HOMO_ = −4.513 eV) energy with the electron delocalisation over the *HO-Ar-O-Ar-* moieties and LUMO (E_LUMO_ = −0.810 eV) energy with the electron distribution over the *HO-Ar-OH-C-* moieties, respectively. However, in the case of volasertib, the electrons were found to distribute over the *-HN-CO=Ar-NH-*pyrimidine ring*-NC=O* moieties in the HOMO orbital, whereas, in LUMO orbitals, electrons were delocalised over *O=C-N-*pyrimidine ring*-NH-Ar* moieties [24]. Furthermore, GaussSum 3.0.2 was employed to visualise the fragments of orbitals within the system by calculating the energy of electron spins per unit volume. The density of states (DOS) investigated the hierarchy of electron contributions of the orbitals that participated in chemical interactions. The DOS spectra worked on Gaussian curves, consisting of molecular orbital information (Figure 3). The DOS expostulated the projection of molecular orbitals in the molecules that demonstrated the occupied (red line) and unoccupied (green line) orbitals [25,26].

#### 2.1.2. Evaluation and Analysis of Chemical Descriptor

The chemical descriptors explain the chemical reactivity of the molecules. According to Koopmans’s theorem, the transfer of electrons takes place from the HOMO energy (E_HOMO_) level to the LUMO energy (E_LUMO_) level, which is represented by ionisation potential and electron affinity, respectively [27,28,29]. Amongst the phlorotannins, PtB demonstrated a significant E_HOMO_ value (ionisation potential) of −4.513 eV and an E_LUMO_ value (electron affinity) of −0.810 eV, indicating its easy electron donating and electron accepting capabilities when compared to volasertib, which showed the higher values of E_HOMO_ (−5.192 eV) and E_LUMO_ (−0.970 eV), respectively [30]. Furthermore, hardness indices measure the stability of molecules, whereas softness indexes show molecular reactivity. The hardness and softness indices predicted by us were 1.851 eV and 0.540 eV for PtB, whereas they were found to be 2.111 eV and 0.473 eV for volasertib, respectively [31,32]. These values of hardness and softness indicate that PtB is chemically less stable and more reactive than the standard drug volasertib. Furthermore, as shown in Table 1, BSSE calculations were performed to eliminate the basis set superposition error and the intramolecular interactions among all of the phlorotannins. The BSSE study investigates the energy error caused by intramolecular interactions in chemical reactions that restrict intermolecular forces during DFT calculations. The chosen basis set overlaps with a surrounding atom, which has an impact on the minimisation process [33,34]. Overall, our prediction and study demonstrated that PtB displayed higher stability and bioactivity, as it has a reduced energy gap (E_HOMO_-E_LUMO_), less hardness, and more softness compared with the other phlorotannins when compared to volasertib [35,36]. As a result, PtB may become increasingly chemically reactive as it approaches the target protein (PLK-1). Table 1 displays the computational values of all DFT parameters predicted in our study.

#### 2.1.3. Evaluation and Analysis of Molecular Electrostatic Potential 

The molecular electrostatic potential (MEP) has a vital role in explicating the possible reactivity region of the molecule. The MEP illustrates the electron density cloud surrounding the nuclei of the molecule and also defines the non-covalent interaction and hydrogen bonding that are involved in chemical reactivity. The MEP is demonstrated in red and blue colours that indicate nucleophilic and electrophilic regions [37]. All the selected phlorotannins showed nucleophilicity ranges from −0.104 × 10^−2^ to −9.690 × 10^−2^ au and electrophilicity ranges from +0.104 × 10^−2^ to +9.690 × 10^−2^ au, respectively (Table 1). Amongst them, PtB had both the red and blue regions with calculated values from −0.104 × 10^−2^ to +0.104 × 10^−2^, respectively (Figure 4). As shown in the MEP map, the nucleophilic region resided on the oxydibenzene ring O_3_, O_15_, and O_16_ atoms, whereas the polyphenol ring having H_61_, H_63_, H_64_, H_65_, and H_69_ atoms was covered by the electrophilic region. Similarly, volasertib revealed the nucleophilic region covered by O_1_, and O_3_ atoms of benzamide as well as a piperazine ring, whereas the electrophilic region expanded on the cyclopropylepiperazine ring with the values −7.462 × 10^−2^ au (nucleophile) and +7.462 × 10^−2^ au (electrophile), respectively [38].

### 2.2. Evaluation and Analysis of Molecular Docking 

**Figure 5:**
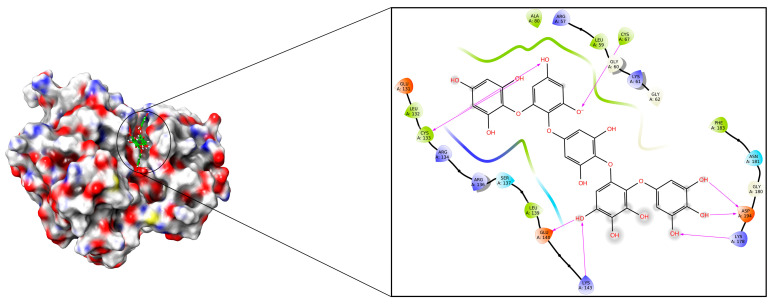
Docking poses along with molecular interactions of PtB within the binding pockets of the 2YAC receptor.

After optimising the structures and chemical reactivity by DFT studies, molecular docking was performed for phlorotannins against the polo-like kinase 1 (2YAC) receptor protein using the GLIDE module 12.5 version of Schrodinger 2020-4 LLC, New York, NY, USA. To rank the ligand poses, the docking scores (kcal/mol) were used as a measure of binding affinities. Our docking results revealed that the phlorotannins were suitably able to fit on the binding pocket site of the PLK-1 protein. As depicted in Table 2, the phlorotannin compounds (**1**–**15**) exhibited binding energies ranging from −7.665 to −5.172 kcal/mol. After an analysis of the results, we obtained a PtB ligand with the lowest binding energy (−7.665 kcal/mol) that was perfectly fit by occupying an ATP-binding cleft within PLK-1. PtB exhibited a binding pocket site of the catalytic domain that had a serine/threonine kinase by forming several molecular interactions like hydrogen bonds with Cys67, Cys133, Glu140, Lys143, Lys178, and Asp194 as well as hydrophobic interactions with Arg57, Leu59, Gly60, Lys61, Gly62, Ala80, Glu131, Leu132, Arg134, Arg136, Ser137, Leu139, Gly180, Asn181, and Phe183 amino acid residues of the PLK-1 receptor. The protein–ligand interaction analysis revealed that the PtB shared hydrogen bonds (donor-acceptor-donor feasibility) with Cys133 and Asp194 amino acids along with further binding with the thiol group of the Cys67 amino acid that participated in the enzymatic reaction [39]. Furthermore, PtB was interconnected by hydrogen bonds with Glu140, Lys143, and Lys178 amino acid residues. Additionally, volasertib as a reference drug has been developed as an antagonist of PLKs and is currently in the I/II phases of clinical trials [40,41]. Volasertib had released −5.172 kcal/mol energy after getting a knot within the binding pockets of the 2YAC by exhibiting hydrogen bonds with Cys67, Cys133, and Arg136; hydrophobic interactions with Arg57, Phe58, Leu59, Gly60, Lys61, Gly62, Gly63, Ala80, Glu131, Leu132, Arg134, Arg135, Glu140, Lys178, Gly180, Asn181, Phe183, Gly196, Leu197, and Thr214; Π-cation interactions with Arg136, as well as salt-bridges with Asp176, and Asp194 amino acid residues, respectively. The best poses in 2D and 3D presentations have been displayed in Figure 5. As per the results obtained, PtB binds effectively within the pocket areas of the protein as compared to volasertib. Thus, it showed reliable molecular interactions with the targeted receptor. Combining these results, PtB complexed with PLK-1 was further subjected to an MD simulation study to better grasp the precise binding stability of our proposed molecule. 

### 2.3. Evaluation and Analysis of Molecular Dynamic (MD) Simulation 

MD simulation is used to study the stability of ligand–protein complexes over a specified time period. Following the DFT and docking studies, a simulation was performed for 100 ns of trajectory to understand the structural dynamics and functional processes of protein–ligand-bound complexes as well as to illuminate the dynamics of the ligand’s binding prototype with the protein in the explicit solvent medium [42,43]. In the present study, the PtB-2YAC complex pose was selected for MD simulation by using Desmond (Maestro version 12.6.144 Schrödinger 2020-4 LLC, New York, NY, USA) software. In addition to ligand characteristics like the radius of gyration, solvent-accessible surface area (SASA), molecular surface area (MolSA), and polar surface area (PSA), the complex’s parameters such as root mean square deviation (RMSD) and root mean square fluctuation (RMSF) are investigated and compared with the reported values as well as a range of fluctuations.

#### 2.3.1. RMSD and RMSF Studies

**Figure 6:**
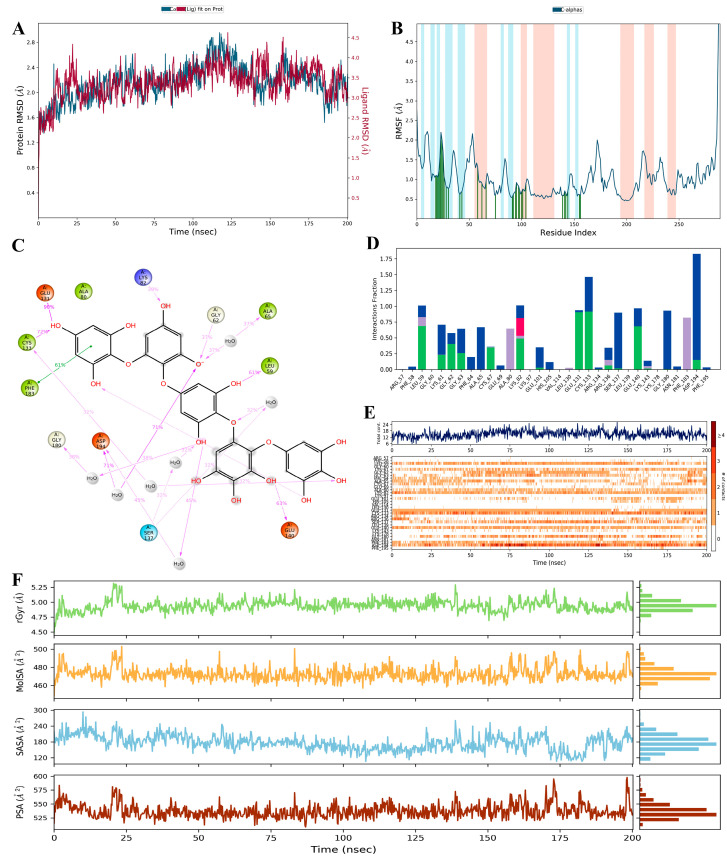
(**A**) RMSD graph of the PtB (red) and 2YAC protein (blue); (**B**) RMSF graph of the 2YAC protein (cyan: β-strand, orange: helices, green: H-bond); (**C**) PtB interactions with protein residues; (**D**) histogram of interaction amino acids of the 2YAC protein (green: H- bond, Blue: water bridges, Pink: ionic bond, cyan: hydrophobic interaction); (**E**) fingerprints of PtB and 2YAC contacts (blue: amino acids contribution, orange: amino acid interactions); and (**F**) PtB properties, e.g., rGyr (green), MolSA (yellow), SASA (light blue), and PSA (dark red). Plots A and B are the RMSD and RMSF presentations among the triplicate runs (Supplementary Information, Figure S1).

RMSD plays a key role in explaining the overall stability of formed complexes. In our study, we analysed the data using the SID (Simulation Interaction Diagram) panel that displayed the RMSD values as 1.394–4.630 Å and 1.167–2.956 Å for ligand and protein in the PtB-2YAC complex (Table 3). PtB showed the rotational movements with RMSD 2.620 Å in the enzymatic site from the initial frame to 10 ns but translated from its original location, having conformational changes with RMSD 3.230 Å–4.340 Å from 11.000 ns–22.200 ns time duration, followed by obtaining equilibrium at the end of the simulation. The stability of the PtB-2YAC complex is shown in the graph (Figure 6A). On the other hand, RMSF assists in analysing the certain positional changes in the amino acid residues of the protein that impart ligand–protein complex equilibrium throughout the simulation. Less fluctuation of amino acids indicates better stability, whereas poorer stability refers to higher fluctuation. During the 200 ns simulation, the amino acids in proteins did not show fluctuations with noted RMSF values ranging from 0.454 to 6.985Å (Table 3). Apart from this, slight conformational changes had been observed in the enzymatic site. PtB had attained stability within the 2YAC protein by forming hydrogen bonds with amino acid residues of the helices (orange) and β-strands (cyan) regions, which are displayed in the graph (Figure 6B) [44].

#### 2.3.2. Ligand Properties Analysis

##### Radius of Gyration (Rg) Study

The radius of gyration (Rg) determines the compactness of the ligand that twirls in the enzymatic sites of the protein. The average value of Rg for PtB in the complex was calculated from 4.588 to 5.316Å throughout the 200 ns simulation time (Table 3). Initially, for a 14 ns time period, the Rg value of PtB decreased but after that, it attained stability (equilibrium) for the whole simulation process. The higher fluctuations of Rg signify less affinity of the ligand with the protein, whereas the PtB shows lower Rg values, exhibiting compactness or stability with the protein throughout its full-time trajectory [45]. 

##### Solvent-Accessible Surface Area (SASA) Study

The SASA refers to the solvent accessibility that provides the quantitative calculation of ligands with protein-implicit water molecules. In our experiment for 200 ns MD simulation, we found the SASA values from 105.693 to 293.441Å^2^ (Table 3). As shown in Figure 6F, the graph indicated that SASA values of the PtB-2YAC complex fluctuated from the initial value to a 293.441 Å^2^ value for a 10.400 ns time period and after that, we also observed that the SASA values decreased gradually and then achieved stability within the enzymatic site of the protein over the full simulation time. 

##### Molecular Surface Area (MolSA) and Polar Surface Area (PSA) Studies

The MolSA measures the water solvent area that ligand molecules acquire. In the MolSA study, the surface area is measured by applying a 1.4 Å probe radius, which is approximately equivalent to the van der Waals surface area and radius of one water molecule. During our study, the MolSA of PtB was calculated from 457.037 to 503.076 Å^2^ throughout the MD simulation (Table 3). The MolSA fluctuated by PtB from the initial frame to 503.076 Å^2^ for 23.400 ns time duration. Furthermore, PtB achieved the equilibrium with the interacted amino acids of said protein and decreased the surface area of the PtB-2YAC complex to the end of the simulation [46]. In addition, the PSA measures the solvent surface area that is produced by polar groups such as nitrogen, and oxygen atoms of the ligand during the simulation. In the current study, the PSA for polar atoms of PtB that interacted with amino acids in the active site was estimated from 508.501 to 597.879 Å^2^ in a 200 ns simulation (Table 3). Initially, the polar atoms of PtB deviated up to 560.750 Å^2^ for 2 but the polar surface area was decremented, and PtB bound with amino acids of protein via intermolecular interactions in the enzymatic site at the end of the simulation [47].

##### Ligand–Protein Interactions Studies

The formation of hydrogen, hydrophobic, ionic, and water-mediated bonds demonstrates the ligand–protein interaction. In this study, we have analysed the contacts of the stable PtB-2YAC complex by displaying the histogram (Figure 6D). For the PtB and 2YAC contacts in our 200 ns simulation, we found that Leu61 (59%), Gly62 (37%), Lys82 (39%), Glu131 (90%), Cys133 (72%), and Glu140 (63%) were interconnected to the PtB through hydrogen bonds as well as water-mediated bonds with Cys133, Ser137, Gly180, and Asp194 amino acid residues of the protein. On the other hand, the aromatic ring of the Phe183 (61%) displayed a hydrophobic (π-π stacking) interaction with the phenol ring of the PtB with a 3.67 Å bond distance, but the π-π stacking interaction is not prevalent as an H-bond (Figure 6C). Phenylalanine is a non-polar amino acid that consists of a conjugated benzyl ring, which shows a hydrophobic nature [48]. The distance between amino acids of 2YAC and PtB in the complex was calculated as 2.8 Å for the hydrogen bond, which was significant for the stability of the ligand in the enzymatic site [49]. 

### 2.4. Evaluation and Analysis of MMGBSA Study

To estimate the free binding affinity of the PtB-2YAC complex, the molecular mechanics-generalised born surface area (MMGBSA) method was utilised. Table 4 depicts the various components of binding energy such as Gibb’s free energy, columbic, hydrogen bonds, lipophilic, generalised-born solvation, and van der Waals energies. The PtB-2YAC complex was visualised on a 2D LID workspace in which PtB formed a total of six hydrogen bonds with Leu59, Lys61, Cys67, Glu131, Cys133, Glu140, and Asp194 amino acid residues. The PtB effectively binds (ΔG_bind_ = −67.915 Kcal/mol) through hydrogen bonds, exhibiting significant stability in the enzymatic site of the PLK-1 protein [50].

The current study provided evidence that phlorotannins, which are prevalent in brown edible algae, have the ability to control the maintenance of mitosis during cell division and, as a result, increase the death of cancer cells. Calculated (predicted) values revealed that most of the selected phlorotannins were in good agreement to pass out in drug-likeness parameters; hence, phlorotannins may be promising options as therapeutic agents against cancer. Moreover, the MD simulation results also revealed that amongst the selected phlorotannins (**1**–**15**), PtB was stable within the binding pockets of the PLK-1 protein at the enzymatic (serine/threonine kinase) site. It does not leave the binding site during the entire trajectory time. In addition, we established the validation of our molecular docking by doing MMGBSA analysis that was also in a significant mode and confirmed the overall stability of the PtB-2YAC complex. Finally, we firmly believe in our results that phlorotannins, most importantly PtB, could act as promising inhibitors of the PLK-1 protein. Further, in vitro and in vivo experimental validations are needed to support our predictions to combat different types of cancer via PLK-1 modulation.

## 3. Materials and Methods

### 3.1. Density Functional Theory (DFT) Calculations

The Gaussian 09W programme is used to model the theoretical structures of phlorotannins. The optimised structures were evaluated using the GaussView 6.0.16 package by using B3LYP functions with a 6–31 G basis set. The DFT calculations explored electronic properties such as frontier molecular orbitals (HOMO and LUMO), energy, and energy gap (E_gap_). Some reactive descriptors have also been included such as ionisation energy (IE), electron affinity (EA), hardness (ƞ), and softness (σ) (Equations (1)–(4)). Additionally, the basis set superposition error (BSSE), as well as the states of orbitals of all the optimised phlorotannin, were calculated and plotted by using the counterpoise (CP) method and GaussSum 3.0.2 software, respectively [51,52].

IE = −E_HOMO_ (1)

EA = −E_LUMO_ (2)

η = (IE − EA)/2(3)

σ = 1/η(4)

### 3.2. Ligand Preparation

**Figure 7:**
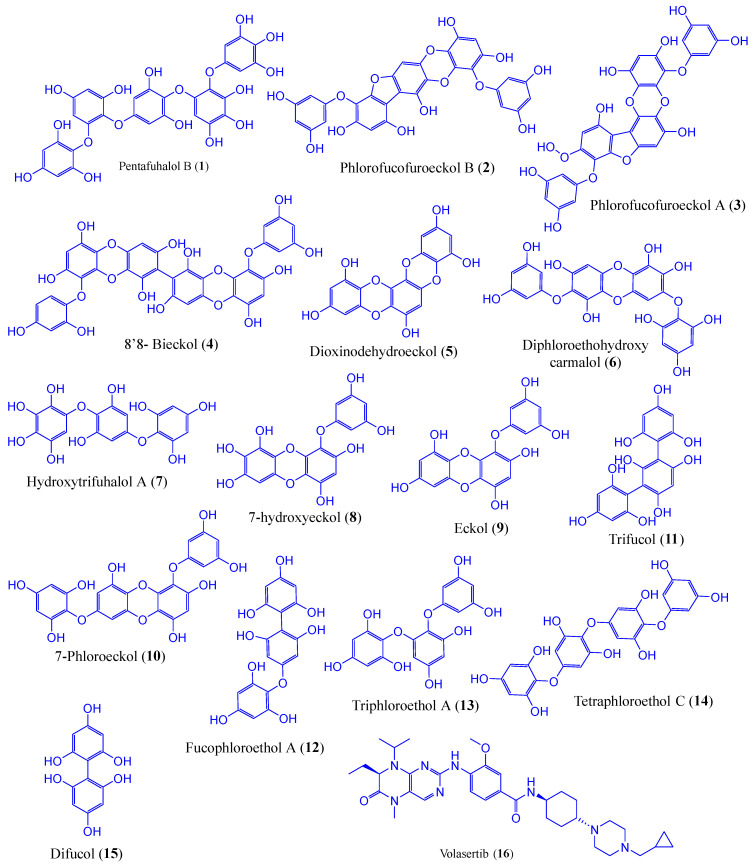
The chemical structures of phlorotannin compounds (**1**–**15**) and the control drug, volasertib (**16**).

In this study, we constructed a library of 15 phlorotannins (Figure 7) that are abundantly found in edible brown algae. The chemical structures in sdf (spatial data file) format were obtained from PubChem (pubchem.ncbi.nlm.nih.gov/, accessed on 28 December 2022). The two-dimensional (2D) chemical structures were inserted from the workspace after drawing with the 2D sketcher tool to generate the ligands for the docking study using the LigPrep tool (Maestro 12.5v Schrödinger 2020-4 LLC, New York, NY, USA). The LigPrep tool turned 2D chemical structures into three-dimensional (3D) ones to reduce and then rectify the geometrical structure using an OPLS3e force field. The possible tautomers, as well as the preserved chirality of 3D structures, were created using the Epik pH 7.0 +/−2 by default ionisation conditions [53]. 

### 3.3. Protein Preparation and Grid Generation

The PLK-1 protein of 36.39 kDa molecular weight has a single chain (Chain A) of 311 amino acid sequences with a resolution of 2.20 Å. The 3D structure of PLK-1 (PDB ID: 2YAC) was retrieved from the RCSB Protein Data Bank (www.rcsb.org/structure/, accessed on 28 December 2022). The co-crystal structure of 2YAC contains a native ligand, namely NMS-P937, which shows excellent interaction in the enzyme’s active region at Glu131 and Cys133 amino acid residues of the said protein. The amide functionality of the ligand makes H-bonds with the amino acids Lys82 and Asp194, along with the formation of a sandwich structure with the amino acids Cys67 and Phe183. This ligand also inhibits the PLK-1 enzyme with an IC50 of 2 nM, which is more than 1000-fold more specific for PLK-1 than PLK-2 and PLK-3, according to one study. It is very effective against the target PLK-1 and has good solubility and pK characteristics, whether administered in vivo or orally. Also, the native ligand is found to be a PLK-1 inhibitor taken orally and studied in clinical trial phase I [54,55]. To assist in the protein preparation for molecular docking, we employed the protein preparation wizard (Maestro 12.5v Schrödinger 2020-4 LLC, New York, NY, USA). The protein was pre-processed by removing heteroatoms and water molecules, aligning the bond order, and adding hydrogen atoms. Prior to protein minimisation, all hydrogen bonds were optimised using the PROPKA 7.0 pH method, and then the OPLS3e force field was used to minimise the protein structure. To perform molecular docking, we also created a grid over the native ligand NMS-P937, which was bound within the catalytic site (X = −9.2, Y = 1.2, Z = 7.59) of the protein. Later on, the default van der Waals radius with a scaling factor of 1.0 Å and a 0.25 partial cut-off was selected by using the receptor grid generation (Maestro 12.5 v Schrödinger 2020-4 LLC, New York, NY, USA) tool [56,57]. 

### 3.4. Molecular Docking and Pose Visualisation 

The Glide (Maestro 12.5 Schrödinger 2020-4 LLC, New York, NY, USA) molecular docking tool was used to undertake structure-based virtual screening of candidate inhibitors against polo-like kinase-1 (PLK-1). During the molecular docking approach, the catalytic site of the targeted receptor was rigid and the flexible technique was utilised for the ligand to perform molecular docking with standard precision (SP) under a 0.15 Å partial cut-off along with a 0.80 Å van der Waals scaling factor to soften the potential for the non-polar part of the ligand. Moreover, the docking mechanism generates the conformations of the ligands and predicts the possible inhibitor for the PLK-1 target based on the lowest binding scores. Furthermore, the ligand–receptor complex was visualised using a pose viewer interface [58,59]. The docking scores were calculated as the best scores among triplicate runs.

### 3.5. Molecular Dynamic Simulations

The MD simulation is used to examine the dynamic properties of the protein–ligand complex in order to optimise its stability. The Desmond package (Schrödinger 2020-4 LLC, New York, NY, USA) was employed for evaluating the stability of PtB complexed with the 2YAC receptor for 200 ns of trajectory time. The complex (PtB-2YAC) was developed as a TIP3P water model in an orthorhombic box shape with a defined 10.0 × 10.0 × 10.0 Å^3^ three-dimensional shape of periodic boundary condition. The complex systems were minimised by enforcing the OPLSe force field with the system builder panel after being neutralised by 10 Cl^-^ charges with a 0.15 M salt concentration. The entire system of complexes was loaded from the workspace to run the MD simulation (triplicate runs) using the Nosé–Hoover and Martyna–Tobias–Klein methods with 1.0 ps and 2.0 ps relaxation times under the NPT (constant number of particles, pressure, and temperature) isothermal-isobaric ensemble condition [60,61]. The r-RESPA integrator assessed the electrostatic interactions along with the binding force in between the atoms with a short-range coulombic force having a cut-off of 9.0 on each 4.8 ps time interval [62]. Finally, the complex simulation trajectory was studied using the RMSD, RMSF, and radius of gyration parameters.

### 3.6. MMGBSA Analysis

The molecular mechanics-generalised born surface area (MMGBSA) is a prominent method to calculate the free binding energy of a ligand to a particular protein target. To validate the complex stability, the prime MMGBSA 3.0 version tool (Schrödinger 2020-4 LLC, New York, NY, USA) was used for obtaining the trajectory of PtB against the 2YAC protein. The binding free energy was estimated through the thermal_mmgbsa.py command, which followed the MMGBSA equations.

ΔGbinding = ΔEMM + ΔGsolvent + ΔGSA(5)

where ΔE_MM_ is the difference between the total energy of a complex and the summation of ligand and receptor energies, ΔG_solvent_ is the difference between the total solvation energy and the energy summation of a ligand with the receptor, and ΔG_SA_ is the difference between the total energy of the surface area and the summation of energy surface area for the ligand and receptor [63,64].

## 4. Conclusions

PLK-1 is a crucial component of the PLK family, which leads to various cancers including lung cancer. PLK-1 is an important regulator and the most suitable target to treat mitosis for the development of a small molecule antagonist. In this study, we found that PtB showed the lowest binding affinity with key residues Cys67, Cys133, and Arg136 of the ATP domain in the target protein (PLK-1) as compared to volasertib, and the MD simulation explored the stability of the ligand with the protein implicit water system. The ligand was found to be stable and displayed minimum deviation and rigidity in the cavity and minimal fluctuation in amino acids throughout the simulation. Observation of Rg showed that PtB firmly moved with amino acids in the cavity as well as the solvent-accessible areas such as MolSA, and SASA, whereas PSA showed equilibria at the end of the simulation around the whole system. Furthermore, the MMGBSA calculation also validated the PtB-2YAC complex by exerting good free binding energy. We also evaluated the chemical reactivity by the DFT study, which predicted the higher reactivity of PtB as compared to volasertib since PtB demonstrated a lower HOMO-LUMO gap, which demonstrates PtB to be softer as compared to volasertib. Hence, our findings in this study demonstrated PtB as a potent antagonist for the PLK-1 target. Further, this study may be helpful in the development of more potent small-molecule-based anti-cancer therapeutics.

## Figures and Tables

**Table 1:** HOMO, LUMO energy, chemical descriptors, and BSSE analysis of phlorotannins (**1**–**15**) and the control drug (**16**), calculated by the B3LYP hybrid function combined with a 6–31 basis set using the DFT method.

Compounds	E_HOMO_ (eV)	E_LUMO_ (eV)	E_gap_ (eV)	IE (eV)	EA (eV)	η (eV)	σ (eV)	MEP (au)	BSSE (Kcal)
**1**	−4.513	−0.810	3.703	4.513	0.810	1.851	0.540	−0.104 × 10^−2^ to +0.104 × 10^−2^	5.404
**2**	−5.315	−0.910	4.405	5.315	0.910	2.202	0.454	−8.301 × 10^−2^ to +8.301 × 10^−2^	3.396
**3**	−5.501	−1.012	4.489	5.501	1.012	2.244	0.445	−7.982 × 10^−2^ to +7.982 × 10^−2^	4.225
**4**	−5.081	−0.452	4.629	5.081	0.452	2.314	0.432	−7.656 × 10^−2^ to +7.656 × 10^−2^	2.684
**5**	−5.316	−0.647	4.669	5.316	0.647	2.334	0.428	−8.071 × 10^−2^ to +8.071 × 10^−2^	2.630
**6**	−5.292	−0.530	4.762	5.292	0.530	2.381	0.419	−9.166 × 10^−2^ to +9.166 × 10^−2^	4.878
**7**	−5.094	−0.308	4.786	5.094	0.308	2.393	0.417	−9.690 × 10^−2^ to +9.690 × 10^−2^	4.144
**8**	−5.279	−0.469	4.810	5.279	0.469	2.405	0.415	−8.195 × 10^−2^ to +8.195 × 10^−2^	4.482
**9**	−5.272	−0.423	4.853	5.272	0.423	2.425	0.412	−7.643 × 10^−2^ to +7.643 × 10^−2^	3.279
**10**	−5.496	−0.603	4.876	5.496	0.603	2.432	0.411	−8.170 × 10^−2^ to +8.170 × 10^−2^	1.068
**11**	−4.987	0.097	5.084	4.987	−0.097	2.542	0.393	−7.903 × 10^−2^ to +7.903 × 10^−2^	4.021
**12**	−5.699	−0.602	5.097	5.699	0.602	2.548	0.392	−8.353 × 10^−2^ to +8.353 × 10^−2^	3.745
**13**	−5.681	−0.520	5.166	5.681	0.520	2.583	0.387	−8.430 × 10^−2^ to +8.430 × 10^−2^	4.298
**14**	−5.514	−0.276	5.238	5.514	0.276	2.619	0.381	−8.594 × 10^−2^ to +8.594 × 10^−2^	1.516
**15**	−5.570	−0.307	5.263	5.570	0.307	2.631	0.380	−7.782 × 10^−2^ to +7.782 × 10^−2^	2.557
**16**	−5.192	−0.970	4.222	5.192	0.970	2.111	0.473	−7.462 × 10^−2^ to +7.462 × 10^−2^	7.918

E_HOMO_ = HOMO energy, E_LUMO_ = LUMO energy, E_gap_ = energy gap, IE = ionisation energy or—E_HOMO_, EA = electron affinity or—E_LUMO_, η = (IE-EA)/2, σ = 1/η, MEP = molecular electrostatic potential.

**Table 2:** The binding energy of phlorotannins (**1**–**15**) with their molecular interactions with 2YAC receptor.

Compounds	* Binding Energy	H-Bonds Interaction	Hydrophobic Interaction	Other Interaction
**1**	−7.665	Cys67, Cys133, Glu140, Lys143, Lys178, Asp194	Arg57, Leu59, Gly60, Lys61, Gly62, Ala80, Glu131, Leu132, Arg134, Arg136, Ser137, Leu139, Gly180, Asn181, Phe183	-
**2**	−7.635	Arg57, Arg134, Cys133, Glu140, Lys178, Asn181	Leu59, Gly60, Lys61, Gly62, Cys67, Ala80, Leu132, Arg135, Arg136, Leu139, Lys143, Gly180, Phe183, Asp194	-
**3**	−6.846	Cys133, Glu140, Lys178, Asp194	Leu59, Gly60, Gly62, Cys67, Ala80, Lys82, Val114, Leu130, Glu131, Leu132, Arg136, Leu139, Lys143, Gly180, Asn181,	Π-Π-Phe183
**4**	−6.729	Cys67, Cys133, Glu140, Asp176,	Leu59, Gly60, Lys61, Gly62, Gly63, Ala80, Lys82,Hie105, Val114, Leu130, Glu131, Leu132, Arg136, Ser137, leu139, Asn181, Phe183, Gly193, Gly196, Leu197	Salt-Lys178
**5**	−6.511	Leu59, Cys133, Glu131, Asp194	Arg57, Gly60, Gly62, Cys67, Ala80, Val114, Leu130, Leu132, Arg134, Arg136, Phe183	Salt-Lys82
**6**	−5.897	Cys67, Arg136, Lys143, Asp194	Leu59, Gly60, Gly62, Ala80, Lys82, Hie105, Val114, Leu130, Glu131, Leu132, Cys133, Leu139, Glu140, Asn181, Gly193	Π-Π-Phe183
**7**	−5.767	Cys67, Cys133, Asp194	Arg57, Leu59, Gly60, Lys61, Gly62,Leu132, Arg134, Arg136, Glu140Lys178, Asn181, Phe183	-
**8**	−5.708	Leu59, Cys133, Asp194	Gly60, Gly62, Cys67, Ala80, Hie105, Val114, Leu130, Glu131, Leu132, Arg134, Arg136, Lys178, Gly180, Asn181,Gly193	Salt-Lys82, Π-Π-Phe183
**9**	−5.656	Cys67, Cys133, Glu131, Asp194	Leu59, Gly60, Lys61, Gly62, Ala80, Lys82, Hie105, Val114, Leu130, Leu132, Arg136, Glu140, Phe183, Gly193, Phe195	-
**10**	−5.489	Leu59, Lys61, Cys67, Cys133, Asp194	Gly60, Gly62, Ala80, Leu130, Leu132, Arg134, Arg136, Glu140, Lys178, Asn181, Phe183	Salt-Arg57
**11**	−5.348	Leu59, Lys133, Asp194	Arg57, Gly60, Lys61, Gly62, Ala65, Cys67, Lys82, Leu132, Arg134, Arg136, Ser137, Leu139, Glu140, Gly180, Asn181, Phe183	-
**12**	−5.142	Cys67, Cys133, Asp194, Lys178, Gly180	Leu59, Gly60, Lys61, Gly62, Ala80, Glu131, Leu132, Arg136, Ser137, Asn181	Π-Π-Phe183
**13**	−4.976	Cys133, Glu140, Asp194	Leu59, Gly60, Lys61, Gly62, Ala80, Lys82, Leu130, Glu131, Leu132, Arg136, Ser137, Leu139, Lys178, Gly180, Asn181	Π-Π-Phe183
**14**	−4.953	Leu59, Cys133, Arg134, Asp194	Arg57, Gly60, Lys61, Gly62, Cys67, Lys82, Leu132, Arg135, Arg136, Ser137, glu140, Lys178, Asn181	Π-Π-Phe183
**15**	−3.087	Glu140, Asp194	Gly60, Lys61, Gly62, Ala65, Cys67, Lys82, Ser137, Leu139, Lys178, Gly180, Asn181, Phe183	-
**16**	−5.172	Cys67, Cys133, Arg136	Arg57, Phe58, Leu59, Gly60, Lys61, Gly62, Gly63, Ala80, Glu131, Leu132, Arg134, Arg135, Glu140, Lys178, Gly180, Asn181, Phe183, Gly196, Leu197, Thr214	Π-cation Arg136, Salt-Asp176, Asp194

* The presented docking scores are the best values among triplicate runs (Supplementary Information, Table S1).

**Table 3:** Calculation stability of the PtB-2YAC complex using the MD simulation method.

Parameters	* PtB-2YAC Complex
RMSD of Protein (Å)	1.167–2.956
RMSD of PtB (Å)	1.394–4.630
RMSF of Protein (Å)	0.454–6.985
rGyr (Å)	4.588–5.316
MolSA (Å^2^)	457.037–503.076
SASA (Å^2^)	105.693–293.441
PSA (Å^2^)	508.501–597.879

* Best calculated values for MD simulation parameters among triplicate runs (Supplementary Information, Table S2).

**Table 4:** MMGBSA calculation for the PtB-2YAC complex using MD simulation trajectory.

Complex	ΔG_bind_	ΔG_coul_	ΔG_H-bond_	ΔG_lipo_	ΔG_GB_	ΔG_vdW_
PtB-2YAC	−67.915	−191.533	−5.742	−11.187	−7.178	−62.129

* All the binding free energy in Kcal/mol, ΔG_bind_ = binding free energy, ΔG_coul_ = coulombic energy, ΔG_H-bond_ = hydrogen bond energy, ΔG_lipo_ = lipophilic energy, ΔG_GB_ = generalised-born electrostatic solvation energy, ΔG_vdW_ = van der Waals energy. These are the best values for MMGBSA calculations among triplicate runs (Supplementary Information, Table S3).

## Data Availability

All the docking and other computational data related to this research work have been generated in our institute with the requisite systems’ help and reported accordingly in the manuscript.

## Supplementary Materials

The following supporting information can be downloaded at: https://www.mdpi.com/article/10.3390/molecules28155853/s1, Figure S1: The triplicate RMSD and RMSF plots of PtB-2YAC complex throughout 200 ns simulation; Table S1: Triplicate molecular docking of phlorotannins compounds against 2YAC receptor; Table S2: The triplicate calculated parameters of PtB-2YAC complex throughout 200 ns simulation; Table S3: The triplicate MMGBSA binding free energy study of PtB-2YAC complex.

## Sample Availability

Samples of the compounds are not available from the authors.
