# Supplementary material for: Pentafuhalol-B, a Phlorotannin from Brown Algae, Strongly Inhibits the PLK-1 Overexpression in Cancer Cells as Revealed by Computational Analysis
Source: Molecules. 2023 Aug 3;28(15):5853. doi: 10.3390/molecules28155853 (PMC10421442; doi:10.3390/molecules28155853)
Supplement: Supplementary file 1 [file molecules-28-05853-s001.zip › molecules-2488550-supplementary.pdf]

Supplementary Information (SI)

Pentafuhalol-B, a Phlorotannin from Brown Algae strongly Inhibit the PLK-1 overexpression in Cancer Cells as Revealed by Computational Analysis

Waseem Ahmad Ansari [1,2,#], Safia Obaidur Rab [3,#], Mohammad Saquib [4], Aqib Sarfraz [1], Mohd Kamil Hussain [5], Mohd Sayeed Akhtar [6], Irfan Ahmad [3] and Mohammad Faheem Khan [1,2,*]

[1]Department of Biotechnology, Era's Lucknow Medical College & Hospital, Era University, Sarfarazganj, Hardoi Road, Lucknow 226003, India
[2]Department of Chemistry, Era University, Sarfarazganj, Hardoi Road, Lucknow 226003, India
[3]Department of Clinical Laboratory Sciences, College of Applied Medical Sciences, King Khalid University, Abha, Saudi Arabia
[4]Department of Chemistry, University of Allahabad, Prayagraj 211002, India
[5]Department of Chemistry, Govt. Raza P.G. College, Rampur, M. J. P. Rohilkhand University, Bareilly 244901, India
[6]Department of Botany, Gandhi Faiz-e-Aam College, Shahjahanpur-242001, UP, India
[#]Equal contribution as the first author

***Corresponding Author**
Mohammad Faheem Khan
Department of Chemistry & Department of Biotechnology
Era University, Sarfarazganj, Hardoi Road, Lucknow 226003, India
Mobile: +91 7800737252; Email: faheemkhan35@gmail.com

Table S1. Triplicate molecular docking of phlorotannins compounds against 2YAC receptor

Compounds	Binding energy (Kcal/mole)		
**1**	-7.665	-7.665	-7.665
**2**	-7.635	-7.635	-7.635
**3**	-6.846	-6.846	-6.846
**4**	-6.729	-6.729	-6.729
**5**	-6.511	-6.511	-6.511
**6**	-5.897	-5.897	-5.897
**7**	-5.767	-5.767	-5.767
**8**	-5.708	-5.708	-5.708
**9**	-5.656	-5.656	-5.656
**10**	-5.489	-5.489	-5.489
**11**	-5.348	-5.348	-5.348
**12**	-5.142	-5.142	-5.142
**13**	-4.976	-4.976	-4.976
**14**	-4.953	-4.953	-4.953
**15**	-3.087	-3.087	-3.087
**16**	-5.172	-5.172	-5.172

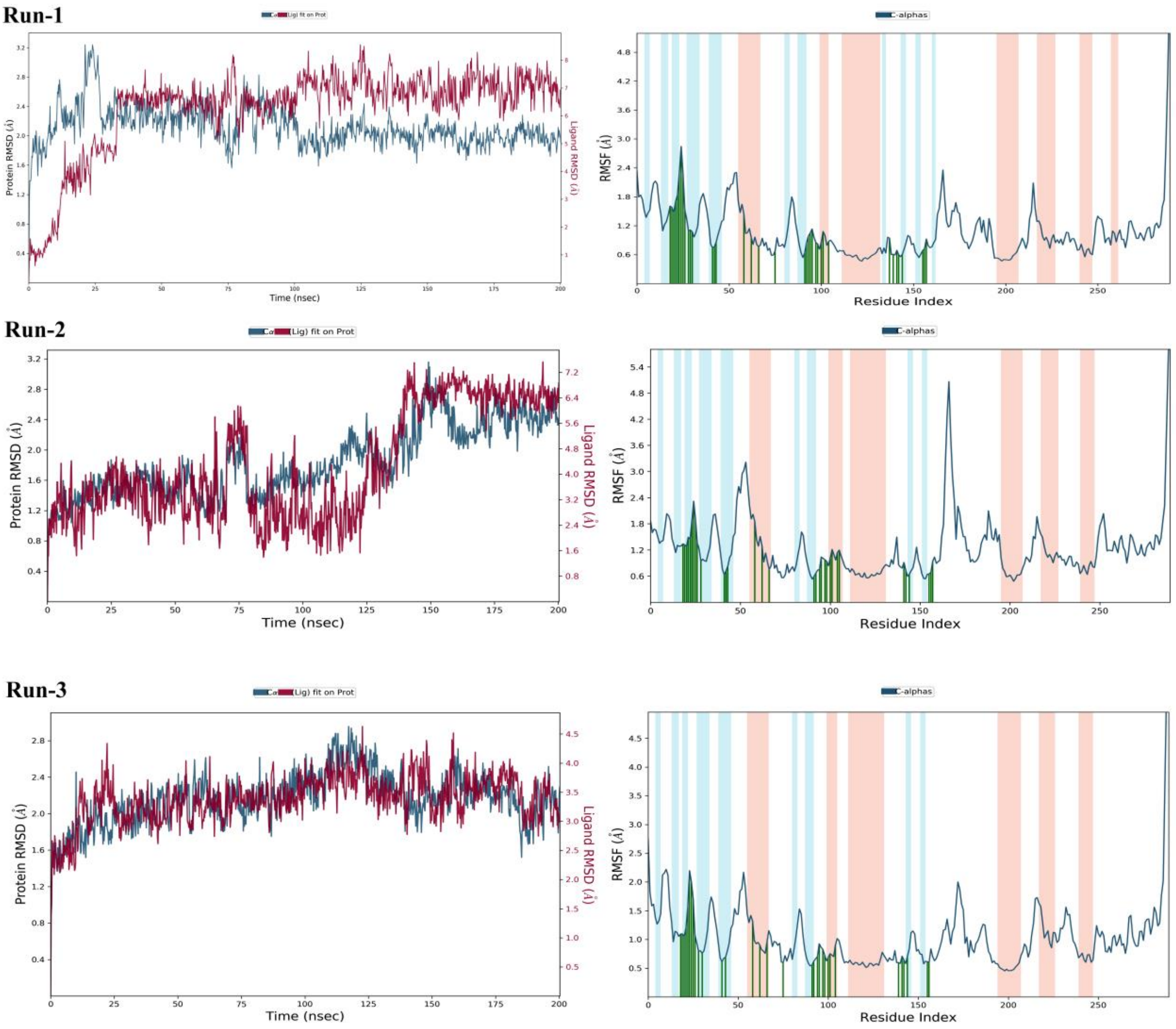

Figure S1. The triplicate RMSD and RMSF plots of PtB-2YAC complex throughout 200ns simulation

Table S2. The triplicate calculated parameters of PtB-2YAC complex throughout 200ns simulation

**Parameters**	**PtB-2YAC complex**		
RMSD of Protein (Å)	1.118 - 3.238	0.794 - 3.159	1.167 - 2.956
RMSD of PtB (Å)	0.606 - 8.550	1.394 - 7.526	1.394 - 4.630
RMSF of Protein (Å)	0.466 - 4.418	0.481 - 7.216	0.454 - 6.985
rGyr (Å)	4.481 - 6.060	4.401 - 5.765	4.588 - 5.316
MolSA (Å^2)	461.249 - 511.531	450.397 - 511.461	457.037 - 503.076
SASA (Å^2)	117.430 - 408.527	149.567 - 404.065	105.693 - 293.441
PSA (Å^2)	528.426 - 604.276	508.704 - 616.063	508.501 - 597.879

Table S3. The triplicate MMGBSA binding free energy study of PtB-2YAC complex

Complex	ΔG_{bind}	ΔG_{coul}	$\Delta G_{H\text{-}bond}$	ΔG_{lipo}	ΔG_{GB}	ΔG_{vdW}
PtB-2YAC	-61.766	-177.145	-6.458	-9.666	-23.537	-57.198
	-59.828	-174.863	-6.347	-9.590	-19.696	-52.335
	-67.915	-191.533	-5.742	-11.187	-7.178	-62.129
